# Cancer-associated fibroblasts induce high mobility group box 1 and contribute to resistance to doxorubicin in breast cancer cells

**DOI:** 10.1186/1471-2407-14-955

**Published:** 2014-12-15

**Authors:** Kamolporn Amornsupak, Tonkla Insawang, Peti Thuwajit, Pornchai O-Charoenrat, Suzanne A Eccles, Chanitra Thuwajit

**Affiliations:** Department of Immunology, Faculty of Medicine Siriraj Hospital, Mahidol University, Bangkok, 10700 Thailand; Department of Surgery, Faculty of Medicine Siriraj Hospital, Mahidol University, Bangkok, Thailand; Tumor Biology and Metastasis, Cancer Research UK Cancer Therapeutics Unit, The Institute of Cancer Research, Sutton, UK

**Keywords:** Breast cancer, Cancer-associated fibroblast, HMGB1, Chemoresistance

## Abstract

**Background:**

Cancer-associated fibroblasts and high mobility group box 1 (HMGB1) protein have been suggested to mediate cancer progression and chemotherapy resistance. The role of such fibroblasts in HMGB1 production in breast cancer is unclear. This study aimed to investigate the effects of cancer-associated fibroblasts on HMGB1 expression in breast cancer cells and its role in chemotherapeutic response.

**Methods:**

Breast cancer-associated fibroblasts (BCFs) and non-tumor-associated fibroblasts (NTFs) were isolated from human breast cancers or adjacent normal tissues and established as primary cultures *in vitro*. After confirmation of the activated status of these fibroblasts, conditioned-media (CM) were collected and applied to MDA-MB-231 human triple negative breast cancer cells. The levels of intracellular and extracellular HMGB1 were measured by real-time PCR and/or Western blot. The response of BCF-CM-pre-treated cancer cells to doxorubicin (Dox) was compared with those pre-treated with NTF-CM or control cultures. The effect of an HMGB1 neutralizing antibody on Dox resistance induced by extracellular HMGB1 from non-viable Dox-treated cancer cells or recombinant HMGB1 was also investigated.

**Results:**

Immunocytochemical analysis revealed that BCFs and NTFs were alpha-smooth muscle actin (ASMA) positive and cytokeratin 19 (CK19) negative cells: a phenotype consistent with that of activated fibroblasts. We confirmed that the CM from BCFs (but not NTFs), could significantly induce breast cancer cell migration. Intracellular *HMGB1* expression was induced in BCF-CM-treated breast cancer cells and also in Dox-treated cells. Extracellular HMGB1 was strongly expressed in the CM after Dox-induced MDA-MB-231 cell death and was higher in cells pre-treated with BCF-CM than NTF-CM. Pre-treatment of breast cancer cells with BCF-CM induced a degree of resistance to Dox in accordance with the increased level of secreted HMGB1. Recombinant HMGB1 was shown to increase Dox resistance and this was associated with evidence of autophagy. Anti-HMGB1 neutralizing antibody significantly reduced the effect of extracellular HMGB1 released from dying cancer cells or of recombinant HMGB1 on Dox resistance.

**Conclusions:**

These findings highlight the potential of stromal fibroblasts to contribute to chemoresistance in breast cancer cells in part through fibroblast-induced HMGB1 production.

## Background

Breast cancer is the most common cancer in females worldwide [1] including Thailand [2]. The standard treatment of breast cancer patients is surgery and chemotherapy. Chemotherapy can be used before (neoadjuvant) or after surgery, with or without other interventions, e.g. radiation or targeted therapy, depending on the subtype and stage of the disease [3]. Unresponsiveness to chemotherapeutic drugs, however, is still the main problem. It has been reported that about 30% of early stage breast cancer patients have a risk of developing drug resistance and cancer recurrence [4]. Resistance is primarily due to the inherent genetic instabilities of cancer cells; however, the resistance acquired during cancer progression and in particular the role of the tumor microenvironment, has also been investigated [5]. A variety of bioactive molecules are secreted by fibroblasts in the tumor microenvironment which can promote tumor growth, metastasis, neoangiogenesis and drug resistance [6–8]. Interactions between cancer cells and stromal fibroblasts reportedly contribute to the chemoresistance of pancreatic ductal adenocarcinoma. The mechanisms described include epigenetic regulation of apoptotic genes in cancer cells [9] and the increased secretion of nitric oxide leading to release of interleukin-1β by the tumor cells that provides protection from anticancer drugs [10].

Moreover, activated fibroblasts in breast cancer have been correlated with the aggressiveness of the disease [11–14] and the induction of acquired chemoresistance [15]. The stromal gene expression pattern has revealed the potential to predict resistance to preoperative chemotherapy with 5-fluorouracil, epirubicin and cyclophosphamide [16]. Collagen type I secreted by fibroblasts can decrease chemotherapeutic drug uptake into cancer cells leading to the regulation of the response to several agents [17]. In addition, critical roles of fibroblasts have been described in tamoxifen resistance via activation of growth factor-related signaling pathways or increased mitochondrial function resulting in an anti-apoptotic effect [18, 19]. Taken together, this evidence suggests that targeting stromal fibroblasts and mechanisms by which cancer-associated fibroblasts are activated may be an emerging novel therapeutic strategy for breast cancer.

High mobility group box 1 (HMGB1) or amphoterin is a chromatin-associated nuclear protein. It has also been recognized as an extracellular "damage-associated molecular pattern" (DAMP) molecule, which has been detected in several diseases including cancer [20]. HMGB1 can be produced by both tumor cells and stromal cells and is released into the extracellular environment from stressed and dying cells [21]. HMGB1 can be released passively from dying tumor cells after chemotherapeutic treatment [22] or following tumor cell lysis by the action of lymphokine-activated killer cells, [23]. In contrast, some studies have reported active secretion of HMGB1 from certain types of cancer [24, 25]. Several chemotherapeutic agents used in the treatment of breast cancer including cyclophosphamide, methotrexate, paclitaxel [22] and doxorubicin [26] induce HMGB1 release into the tumor microenvironment following cell death. Moreover, radiotherapy has also been shown to induce the release of HMGB1 [26]. Finally, it has been shown that host cells, in particular neutrophils and macrophages, are activated by cytokines as part of an innate immune response to cancer cells and actively secrete HMGB1 [27].

Interestingly, factors diffusing from stromal fibroblasts have recently been shown to up-regulate intracellular HMGB1 in lung cancer cells [28]. HMGB1 may then be released from cancer cells during radiotherapy or chemotherapy and act upon surviving cancer cells to promote regrowth and metastasis [29]. Hence we hypothesized that stromal fibroblasts in breast cancer may also play a similar role in chemoresistance through the up-regulation of HMGB1 in cancer cells during chemotherapy-mediated cell death. This study aimed to explore the effect of secreted substances from breast cancer-associated fibroblasts (BCFs) on HMGB1 expression in breast cancer cells and the potential of extracellular HMGB1 to influence chemosensitivity.

## Methods

### Breast cancer cell culture

The human breast cancer cell line MDA-MB-231 was obtained from ATCC-LGC (#HTB-26, Middlesex, UK). Cells were cultured in DMEM (Gibco, Grand Island, NY, USA) supplemented with 10% fetal bovine serum (FBS; Gibco), 100 U/ml penicillin, 100 μg/ml streptomycin (Gibco), and anti-fungal agent. Cells were cultured in a 5% CO_2_ in air incubator at 37°C and passaged by 0.25% trypsin-EDTA when they reached confluence. Cells of more than 90% viability evaluated by trypan blue dye exclusion were used in further experiments.

### Primary cultures of human fibroblasts

Primary cultures of breast cancer-associated fibroblasts (BCFs) were isolated from patients who underwent surgery at Siriraj Hospital, Mahidol University, Bangkok, and non-tumor-associated fibroblasts (NTFs) were isolated in each case from adjacent uninvolved breast tissue. The protocol for tissue collection was approved by the Siriraj Institutional Review Board (si498/2010) and informed consent was obtained from each of the six patients enrolled for this study. All breast cancers were of the hormone receptor positive luminal subtype. Briefly, sterile fresh surgical tissue was placed on ice in DMEM/F12 (Gibco) supplemented with 10X penicillin-streptomycin (1,000 U/ml penicillin and 1,000 μg/ml streptomycin) (Gibco). Tissues were washed 2 to 3 times by 1X phosphate buffered saline (PBS) to remove blood contamination. Tissues were then minced finely using a sterile surgical blade followed by enzymatic dissociation in collagenase type 1A (1,140 U/ml) (Sigma-Aldrich, St. Louis, MO, USA) diluted in DMEM/F12 supplemented with 10% FBS for 2 h at 37°C with agitation every 20 min. Next, tissues were digested in 0.05% trypsin-EDTA (Gibco) in serum-free DMEM/F12 for 10 min. The digestion solution was removed and fragments were washed with DMEM/F12 containing no FBS. The cell suspension was sequentially filtered through 100 μm and 70 μm nylon meshes (BD Biosciences, San Jose, CA, USA) and centrifuged at 2,000 xg for 5 min. The cell pellet was resuspended in complete DMEM/F12 media and cultured in a 25 cm^2^ culture flask (Corning, NY, USA). Cells isolated from tissue samples were incubated in DMEM/F12 media containing 10% FBS for 10–14 days to allow attachment and the formation of colonies; these primary cultures were designated as passage 0. All cultures were kept in a humidified incubator with 5% CO_2_ in air at 37°C. Cells were subcultured when 80% confluent, banked and used for characterization and experimental studies at passages 5–13.

### Immunocytochemistry of BCFs and NTFs

To discriminate BCFs and NTFs from cancer cells, immunohistochemical staining for epithelial CK19 and mesenchymal ASMA markers were performed. The breast cancer cell line, MDA-MB-231 was used as a positive control for CK19 detection. In brief, around 4,000 cells were plated into each well of a 96-well plate and cultured for 24-h to allowed for cell adhesion. Cells were then fixed with 4% paraformaldehyde. Non-specific binding was blocked by incubating cells with 1% BSA in 1X PBS for ASMA detection or 5% FBS in 1X PBS for CK19 detection. Mouse anti-human CK19 antibody (SC-6278; 1:100 dilution, Santa Cruz Biotechnology Inc., Dallas, TX, USA) or mouse anti-human ASMA antibody (A5228, 1:200 dilution, Sigma-Aldrich) was added for 3 h at room temperature. A blocking reagent was used as the negative control in place of the primary antibody. After washing with 1X PBS, goat anti-mouse IgG-Cy3 antibody (#115-166-071, 1:2,000, Jackson ImmunoResearch Laboratories Inc, West Grove, PA, USA) was applied for 1 h at RT. The signals were detected by fluorescence microscopy.

### Collection of fibroblast conditioned-media

Cultures of BCF and NTF were grown in 75-cm^2^ flasks to reach 90-95% confluency in DMEM (containing 10% FBS) which is a suitable media for MDA-MB-231 cells. The conditioned-media (CM) were collected 24 h following addition of fresh complete medium and designated as 24-h CM. CMs were centrifuged at 2,000 g for 5 min to remove cell debris and the suspension stored at -80°C or -20°C until use.

### Scratch wound tumor cell motility assay

MDA-MB-231 cells were cultured in a 6-well plate until approximately 90-100% confluent. A reference line was drawn across the bottom of the plate. A scratch wound was made in the cell monolayer with a sterile 200-μl pipette tip and the culture was then washed three times with serum-free medium to remove the detached cells. The cells were then treated with BCF-CM, NTF-CM or complete medium as a negative control. The scratch wound indicated by the reference line was imaged at the start of the treatment and 6 h later. The cell migration efficiency was determined as a percentage of wound healing calculated by the following formula using three different zones for each condition:


### Real time PCR for HMGB1 mRNA detection

Total RNA was extracted from MDA-MB-231 breast cancer cells using the PerfectPure RNA Cultured Cell Kit (5 Prime; Gaithersburg, MD, USA) as per the manufacturer’s instructions. The OD260/280 and OD260/230 were measured to ensure the quality of extracted RNA. Complementary DNA was synthesized from 1 μg of total RNA using the SuperScript™ III First-Strand Synthesis System for RT-PCR (M-MLV) (Invitrogen, Carlsbad, CA, USA) according to the manufacturer’s instructions. Expression levels of *HMGB1* were determined by SYBR Green-based real time PCR (Roche Applied Sciences, Indianapolis, IN, USA) in a Light Cycler® 480 II machine (Roche Applied Sciences). Optimal primers were designed using the nucleotide database in PubMed and Primer 3 software. Sequences of primers were: *HMGB1* (NM_002128.4): forward primer: 5'-CACTGGGCGACTCTGTGCCTCG-3', reverse primer: 5'-CGGGCCTTGTCCGCTTTT-GCCA-3'. *β-actin* (*ACTB*) served as an internal control to adjust the amount of starting cDNA. The expression of each gene in breast cancer cells was calculated by the 2^-Δ*C*p^ equation. In this case, Δ*C*_p_= C_p_(*HMGB1*) - C_p_(*ACTB*). The expression of *HMGB1* in breast cancer cells treated with fibroblast CM or doxorubicin (Dox) (Pfizer, Perth Pty Ltd, Bentley WA, Australia) compared with that in untreated control cells was calculated by the 2^-ΔΔ*C*p^ equation. In this case, Δ*C*_p_= C_p_(*HMGB1*) - C_p_(*ACTB*) and ΔΔ*C*p= Δ*C*_p_(treated cells) - Δ*C*_p_(control cells).

### Western blot analysis

MDA-MB-231 breast cancer cells were treated with BCF-CM or NTF-CM for 48 hr. Cell suspensions were centrifuged at 2,000 ×g for 5 min in a refrigerated centrifuge. The cell pellets were collected and rinsed in cold 1X PBS twice before lysis in 1X sample buffer containing 50 mM Tris–HCl pH 6.8, 2% (w/v) SDS, 10% (v/v) glycerol, 5% (v/v) β-mercaptoethanol and 0.05% (w/v) bromophenol blue. Cell lysates were boiled for 10 min and centrifuged at 10,000 rpm for 1 min to remove undissolved proteins and cell debris. Cell extracts were then separated by 10% SDS-PAGE and transferred onto PVDF membranes (GE Healthcare, Buckinghamshire, UK). Membranes were blocked in 5% skimmed milk containing TBST (TBS containing 0.1% Tween 20) for 1 h at room temperature. Membranes were then washed 3 times with 1X TBST and incubated with 1:1,000 rabbit anti-human HMGB1 (ab18256, Abcam, Cambridge, CB4 OFL, UK) at 4°C overnight. After washing, membranes were incubated with 1:2,000 goat anti-rabbit IgG-HRP (Abcam) at room temperature for 1 h. The blots were visualized by enhanced chemiluminescence (Thermo Scientific, Rockford, IL, USA). Using 1:10,000 anti-β-actin antibody (sc47778, Santa Cruz Biotechnology Inc.) or 1:10,000 anti-β-actin antibody (ab8226, Abcam) with the suitable HRP conjugated secondary antibody, β-actin protein levels were used as an internal control to confirm equal protein loading. β-actin normalized HMGB1 levels in breast cancer cells without any CM treatment were used as controls.

The same procedure was used for the measurement of extracellular HMGB1 except for the process of sample collection. To measure the amount of HMGB1 released from cells, the media from cells stimulated with either BCF-CM or NTF-CM were collected and concentrated using a 10 kDa cut-off Vivaspin concentrator (Sartorius Stedim Biotech, Goettingen, Germany) at 3,500 ×g. Protein concentration was determined by a Coomassie Plus (Thermo Scientific) assay kit. The same amount of total protein was loaded into SDS-PAGE gels and the procedure for detection of HMGB1 above.

For LC3B autophagy protein detection, cells were treated with 100 ng/ml human rHMGB1 (#1690-HMB-050, R&D Systems, Minneapolis, MN, USA) with or without anti-HMGB1 neutralizing antibody (H00003146-M08, Novus, Littleton, CO, USA) before exposure to Dox for 24 h. Cells were harvested and total proteins were separated and blotted on to the membrane as described above. The rabbit anti-human LC3B (#2775, Cell Signaling Technology, Danvers, MA, USA) was then incubated at 1:2,000 dilution with the membranes at 4°C overnight followed by goat anti-rabbit IgG-HRP (#7044, Cell Signaling Technology; 1:1,200 dilution) at room temperature for 1 h. The blots were visualized as described previously.

### Treatment of MDA-MB-231 breast cancer cells with doxorubicin or conditioned-media

MDA-MB-231 breast cancer cells were cultured in 6-well plates (3 × 10^5^/well) for 48 h, then the growth medium was removed and the cells washed thoroughly with PBS. CM from either BCF or NTF was added to the cells which were incubated for a further 48 h at 37°C in a 5% CO_2_ in air incubator. Negative controls were cultured in parallel in DMEM plus 10% FBS. Cells were then harvested to determine the level of intracellular HMGB1 expression by real time PCR and Western blot analysis. At the same time, the culture media were also collected to investigate the level of extracellular HMGB1. Similarly, 1 and 5 μM doxorubicin Dox (Pfizer) was used to treat MDA-MB-231 breast cancer cells for 48 h. Dox-treated cells were harvested to determine the level of intracellular HMGB1 by real time PCR and the culture medium was also collected to investigate the level of extracellular HMGB1. In addition, cancer cells with or without pre-treatment with fibroblast CMs were then exposed to Dox for 48 h to induce cell death and the release of HMGB1. Cell viability was checked by trypan blue exclusion and the levels of HMGB1 were determined by Western blot analysis as above.

### Response of breast cancer cells treated with recombinant HMGB1 to Doxorubicin

MDA-MB-231 cells were seeded into 96-well plates at 6,000 cells per well and cultured overnight in DMEM + 0.1% FBS. Cells in triplicate wells were then treated with 100 ng/ml rHMGB1 (R&D Systems) in the presence or absence of 10 mg/ml HMGB1-neutralizing antibody (Novus) and then 5 μM Dox or vehicle. Cell viability was measured 24 h later by trypan blue exclusion.

### Effect of CM on Dox sensitivity of MB-231 breast cancer cells

MDA-MB-231 cells were plated at a density of 6,000 cells/well in 96-well plates. These cells were treated for 24 h with CM collected from MDA-MB-231 cells exposed to 5 μM Dox (for 48 h), designated as ‘dead cancer-CM’, with or without anti-HMGB1-neutralizing antibody (10 mg/ml) or isotype-matched control IgG (X0943, Dako, Agilent Technologies, Glostrup, Denmark). Cell viability after exposure to 5 μM Dox was analyzed by erythrosine B dye exclusion and compared with control untreated cells. Three independent experiments were performed.

### Statistical analysis

The values are expressed as mean ± SD. Statistical significance was determined by Student’s t-test. A p-value of equal to or less than 0.05 was defined as statistically significant.

## Results

### Characterization of primary cultures of BCFs and NTFs

**Figure 1:**
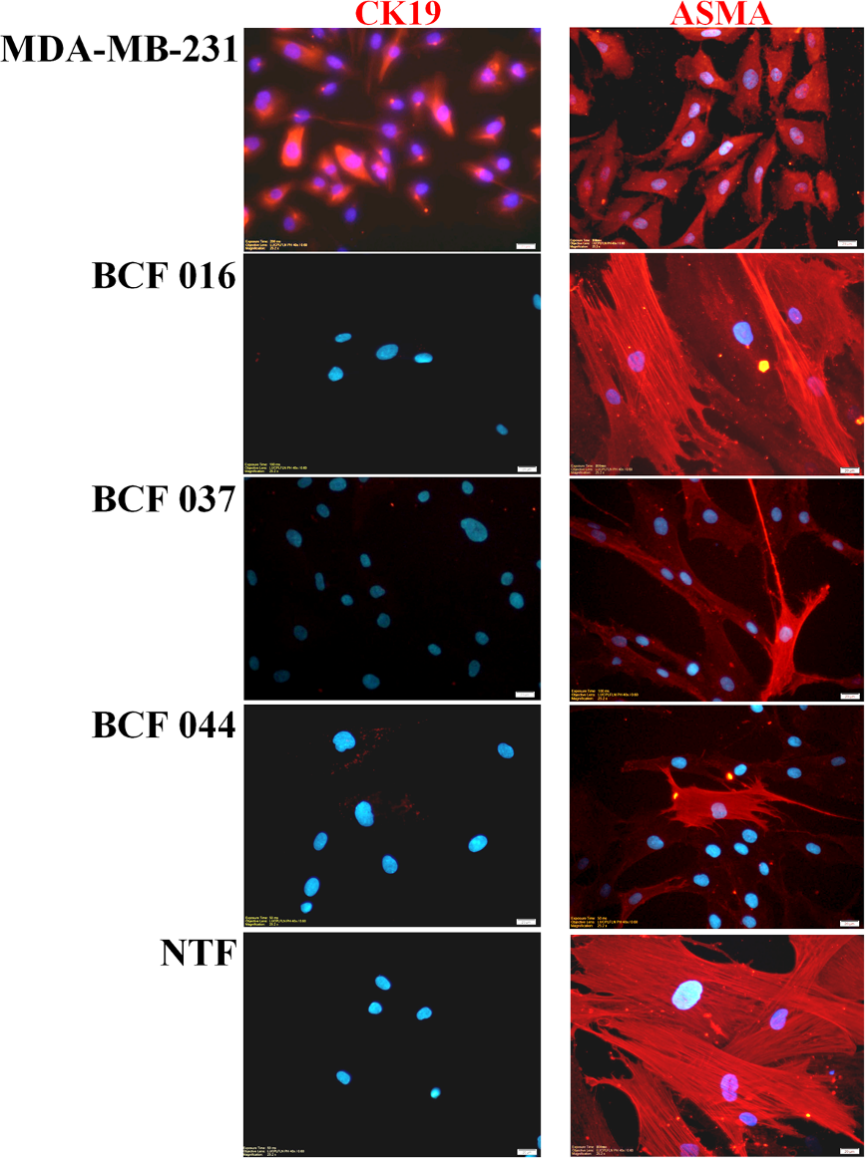
**Immunofluorescent staining of CK19 and ASMA in primary cultures of fibroblasts derived from breast cancers (BCF) and adjacent areas of normal breast from surgical specimens (NTF).** BCF 016, BCF 037 and BCF 044 are derived from different patients, whereas NTF are matched normal tissue fibroblasts pooled from patients 037 and 044. Breast cancer cell line MDA-MB-231 was used as a positive control for CK19. Hoechst (blue) staining shows the nuclei. Original magnification of 400x. Bars represent 20 μm.

**Figure 2:**
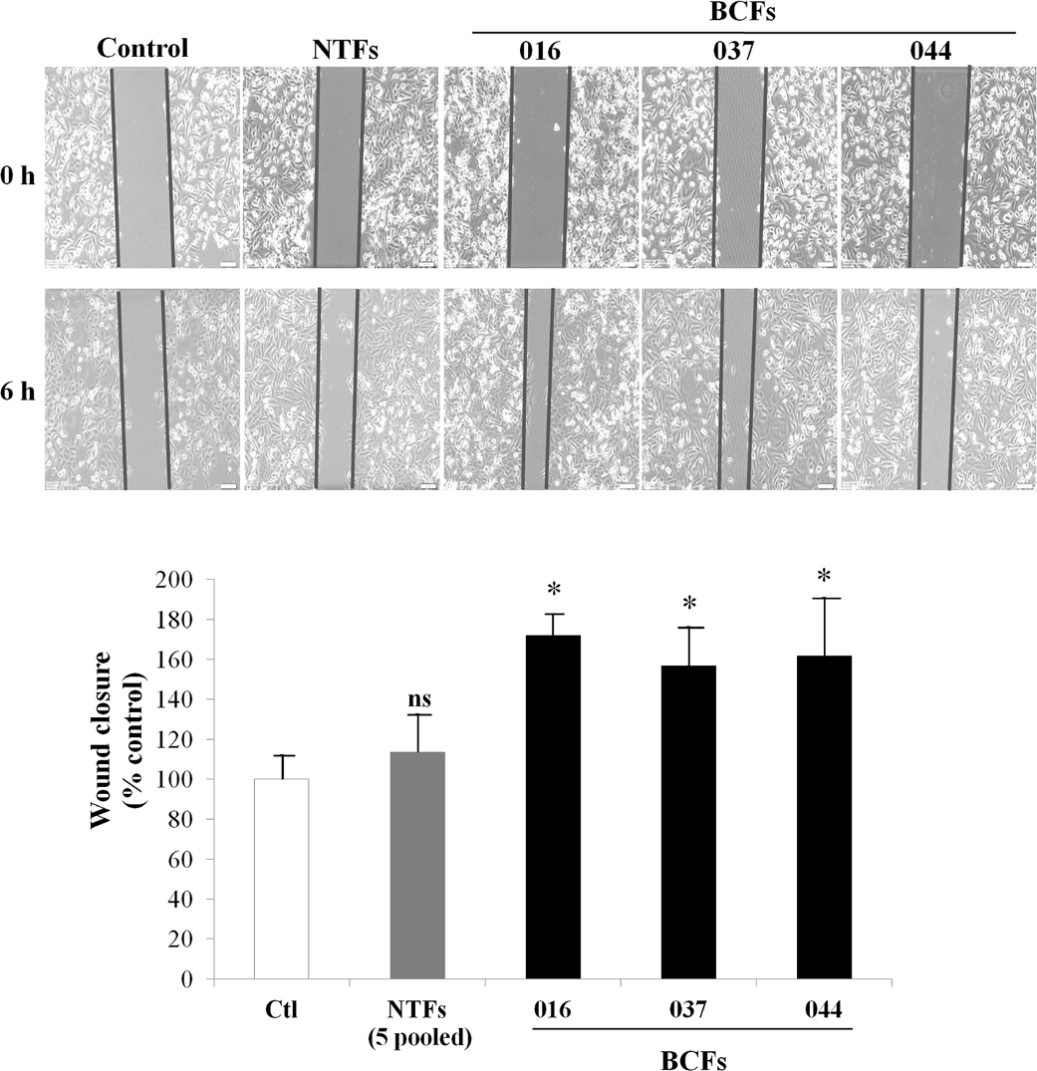
**BCF-CMs enhance MDA-MD-231 cell migration. MDA-MB-231 breast cancer cells were exposed to 3 different BCF-CMs and pooled samples of 5 NTF-CMs and a scratch wound motility assay was performed over 6 h to measure the ability of CM to induce cancer cell migration.** Bar graphs represent mean ± SD of two independent experiments. The migration of cells in control fresh media (Ctl) was set at 100%. * = p-value of less than 0.05 compared to the migration of cancer cells under control conditions. ns = not significance.

BCFs and NTFs were characterized by their expression of the mesenchymal marker, ASMA, and absence of the epithelial marker, CK19. Immunocytochemical staining revealed that all cancer-associated and ‘normal’ breast fibroblasts from six different patients were negative for CK19 compared with the positive control MDA-MB-231 breast cancer cells (Figure 1) and were positive for ASMA. Thus we confirmed that both BCFs and NTFs were mesenchymally-derived cells with no epithelial cell contamination.To ensure that the BCFs were activated and capable of promoting malignant potential, the effects of CM on MDA-MB-231 breast cancer cell migration were tested. The results indicated that the BCF-CM promoted cancer cell migration to a significantly greater degree than NTF-CM (Figure 2). Indeed, NTF-CM had a minimal effect compared with untreated control cells.

### Increased expression of HMGB1 in breast cancer cells treated with fibroblast-derived CM

**Figure 3:**
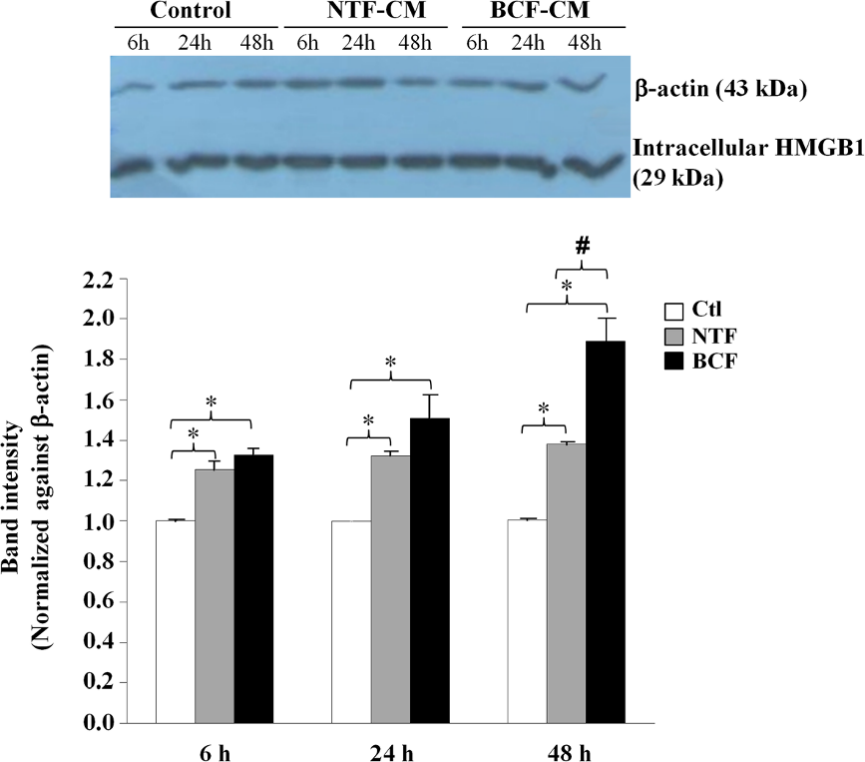
**Western blot analysis of intracellular HMGB1 in MDA-MB-231 human breast cancer cells treated with fibroblast CMs (BCF 044 and NTF 044) for 6, 24, and 48 h.** Cancer cells cultured in fresh medium were used as a negative control. The intensity of each HMGB1 band is shown after normalization against the β-actin internal loading control protein. Bar graphs represent mean ± SD of two independent experiments. * = p-value of less than 0.05 comparing HMGB1 levels in the CM-treated cells with controls at each time point; # = p-value of less than 0.05 comparing HMGB1 levels in BCF-CM-treated cells with NTF-CM treatment.

**Figure 4:**
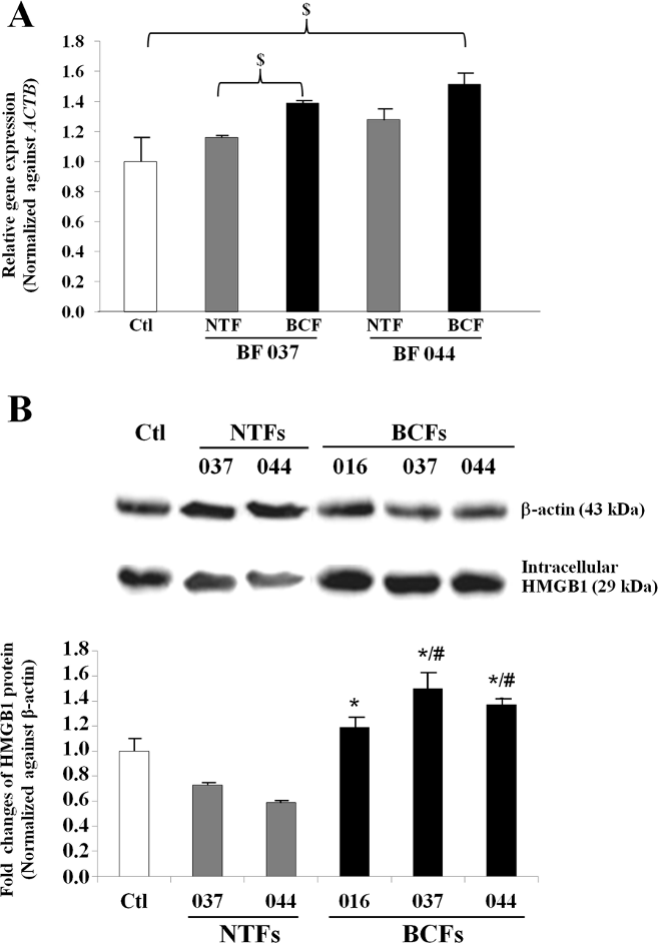
**HMGB1 expression in MDA-MB-231 cells treated with fibroblast CM.** Real time PCR for *HMGB1* expression in MDA-MB-231 cells treated with NTF-CMs and BCF-CMs for 48 h using paired fibroblasts isolated from the same patient.The levels of *HMGB1* transcript **(A)** and protein levels **(B)** are shown after normalization against the internal control β-actin. Controls (Ctl) are cells cultured in fresh medium with no CM treatment. Bars represent the mean ± SD of triplicate experiments. ^$^ = p-value of less than 0.05. * = p-value of less than 0.05 compared to the average HMGB1 of the two NTFs-CM treatment conditions whereas ^#^ = p-value of less than 0.05 compared to HMGB1 of the matched NTF-CM treatment.

BCF-CM significantly induced intracellular HMGB1 protein expression in MDA-MB-231 breast cancer cells as detected by Western blot analysis at all time points tested (Figure 3). The effect was time-dependent and since the greatest differential induction (BCF-CM *vs* NTF-CM) was observed at 48 h, this time period was selected for further studies. As a further quality control, the CMs of BCF and NTF isolated from the same patient were used to treat MDA-MB-231 cells and *HMGB1* gene expression was analyzed by real time PCR. The results showed that BCF-CM induced *HMGB1* mRNA to a significantly greater degree than NTF-CM (Figure 4A). Western blot analysis confirmed that the protein levels of HMGB1 induced by BCF-CM were statistically significantly higher than those induced by patient-matched NTF-CM (Figure 4B). In addition, HMGB1 protein levels in MDA-MB-231 cells treated with BCF-CMs from different patients were consistently significantly higher than those treated with NTF-CMs.

### Cell death induced by Dox promotes expression and release of HMGB1

**Figure 5:**
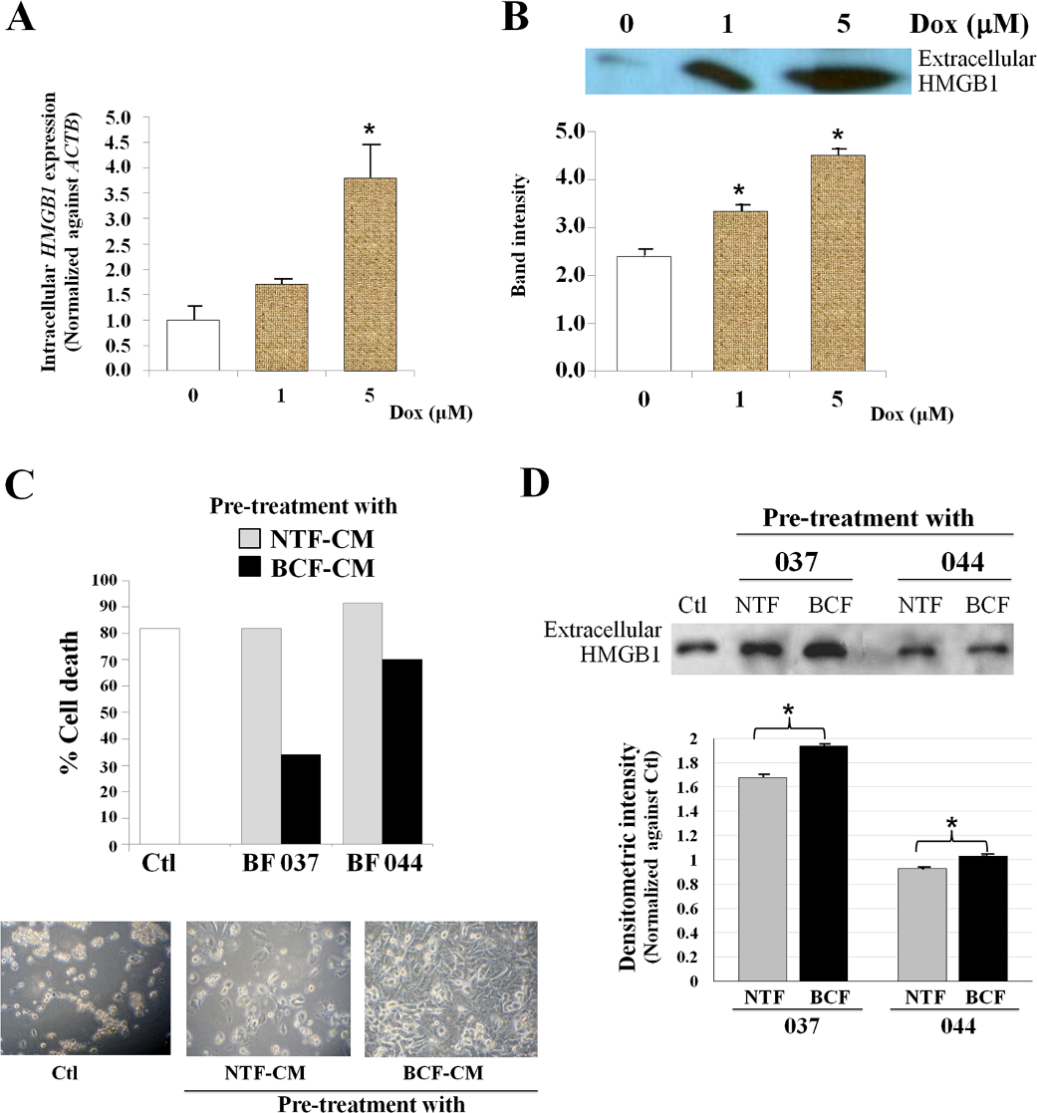
**Dox-induced HMGB1 in MDA-MB-231 cells. (A)** Intracellular *HMGB1* expression was measured by real time PCR. Bars represent mean ± SD of *HMGB1* expression level normalized against *ACTB* and relative to no drug treatment. Three independent experiments were performed. **(B)** Extracellular HMGB1 protein was detected by Western blot analysis in the culture media from cells treated with different concentrations of Dox for 48 h. **(A and B)** *p-value of less than 0.05 compared to the controls without Dox treatment. **(C)** BCF-CM induced resistance to Dox-mediated cell death in MDA-MB-231 breast cancer cells. Bars represent % cell death of each pre-treatment condition and the images show corresponding cell density. **(D)** Culture media from each condition in **(C)** were measured for extracellular HMGB1 by Western blot analysis. Equal amounts of proteins were loaded. Bars represent the mean band intensity (± SD) measured by densitometry. The band intensity of control cultures without CM pre-treatment (Ctl) was set as 1. *p-value of less than 0.05.

Doxorubicin is commonly used in breast cancer treatment and our results using real time PCR showed that this drug could induce intracellular *HMGB1* expression in MDA-MB-231 cells in a concentration-dependent manner (Figure 5A). The maximal level of HMGB1 was induced with 5 μM which was statistically significantly different from untreated controls. Moreover, cancer cells killed by Dox exposure released HMGB1 into the culture media and the level was again increased in a concentration-dependent manner (Figure 5B).BCF-CM-pretreated cancer cell cultures showed less cell death in response to Dox than cells pre-treated with NTF-CM (Figure 5C). In a second study, we found that BCF-CM treated cells also released more HMGB1 than those pre-treated with NTF-CM when treated with equitoxic concentrations of Dox (80% cell death) (Figure 5D). No HMGB1 was detected in the culture media when cell viability was greater than 95% but in contrast, Dox-induced release of HMGB1 was related to the degree of cell death (data not shown).

### Recombinant HMGB1 alters Dox sensitivity via autophagy

**Figure 6:**
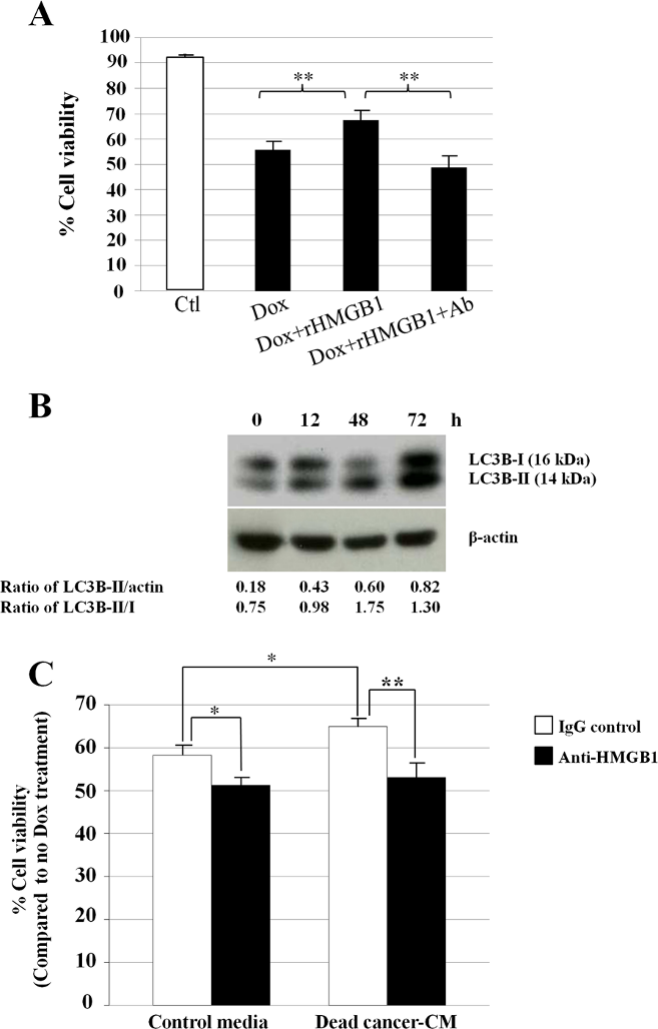
**Effect of recombinant HMGB1 (rHMGB1) on the response of MDA-MB-231 to Dox. (A)** MDA-MB-231 human breast cancer cells were treated with 5 μM Dox with or without addition of 100 ng/ml rHMGB1 and/or anti-HMGB1 neutralizing antibody for 24 h. The % cell viability is shown and the bars represent the mean ± SD of triplicate experimental wells. **(B)** Autophagy-related protein LC3B is induced in MDA-MB-231 breast cancer cells. Densitometric analysis of LC3B-II normalized against the protein loading control β-actin and the conversion of LC3B-I to LC3B-II is shown. **(C)** MDA-MB-231 cells treated with ‘dead cancer-CM’ showed increased viability compared with controls and this effect was attenuated by anti-HMGB1 neutralizing antibody. Bars represent mean ± SD of three independent experiments. * = p-value of less than 0.05 whereas ** is less than 0.01.

MDA-MB-231 cells exposed to Dox together with rHMGB1 showed statistically significantly higher viability than those treated with Dox alone (Figure 6A). This effect was reversed by the addition of an HMGB1 neutralizing antibody. The fact that LC3B-I converts to LC3B-II and levels of LC3B-II increase over time suggests that autophagy occurs in MDA-MB-231 cells treated with rHMGB1 (Figure 6B).

### Dead cancer cell CM attenuates the effects of Dox on MDA-MB-231 breast cancer cell viability in part via HMGB1

Dead cancer-CM, (shown to increase the level of extracellular HMGB1; Figure 5B) also increased the survival of MDA-MB-231 cells during subsequent Dox treatment (Figure 6C) and this effect was reversed by an HMGB1 neutralizing antibody (**p-value = 0.006). The blocking antibody also showed a small but significant reduction in cell viability in the absence of CM (p-value = 0.017).

## Discussion

One of the main reasons for treatment failure in breast cancer is acquired drug resistance. The interaction of tumor cells with their microenvironment has been frequently reported to influence cancer progression and drug resistance [5, 7, 30]. Tumor-associated stromal cells have been shown to protect tumor cells from cell death and the cytotoxic effects of chemotherapeutic drugs [31, 32]. Recently, the impact of cancer-associated fibroblasts on the expression and localization of HMGB1 in lung cancer cells has been demonstrated to operate via the release of diffusible factors from fibroblasts [28]. The extracellular HMGB1 protein behaves as a cytokine, promotes inflammation and participates in the pathogenesis of several disorders in peripheral organs. Extracellular HMGB1 has potential impact in the induction of drug resistance and has been proposed as an immunotherapeutic target to modulate chemotherapeutic responses [22].

Activated fibroblasts in breast cancer tissues have been identified in several reports [11, 15]. In invasive ductal carcinoma, metastatic ability is closely related to the proliferation of fibroblasts [12]. To clearly understand the biological function of fibroblasts in cancer tissues, primary cultures of human fibroblasts are critical. The identity of primary NTFs and BCFs was confirmed here by ASMA positivity [33, 34]. All breast cancer tissues used for isolation of primary cultures of fibroblasts in this study were of luminal subtype and positive for estrogen receptor and/or progesterone receptor. The absence of the CK19 epithelial marker was taken as an indication that the fibroblasts were not cancer cells that had undergone an epithelial-to-mesenchymal transition (EMT). This is supported by the evidence that EMT is most common in basal-like breast cancers [35] and loss of CK19 is rare in hormone receptor-positive breast cancer tissues [36]. ASMA can be used to indicate that the fibroblasts are myofibroblasts but cannot determine their tumor-promoting potential. Cancer-associated fibroblasts have been recognized for their ability to secrete pro-tumorigenic molecules and to promote cancer progression [8, 37]. We used the ability of the BCF-CM to promote breast cancer motility in a scratch wound assay as an indicator of their activated phenotype.

The present study confirmed the effect of fibroblast-derived substances in enhancing HMGB1 expression in human breast cancer cells. Primary cultures of fibroblasts isolated directly from breast cancer tissues (and with patient-matched control cells) were used in preference to established fibroblast cell lines for greater clinical relevance. Cancer cells exposed to fibroblast CM showed increased survival in response to a standard chemotherapeutic agent, doxorubicin. This protective effect correlated with the increased level of HMGB1 released from the cells, was mimicked by recombinant HMGB1 and reversed by the HMGB1 blocking antibody. These observations suggest that cancer-associated fibroblasts should be considered as a possible therapeutic target to attenuate acquired chemoresistance in breast cancer patients via activation of HMGB1.

Interestingly, the CM derived from BCFs induced the production of HMGB1 in cancer cells in a time-dependent manner to a significantly greater degree than the CM from NTFs derived from adjacent non-involved breast tissue in the same patient. These results are in agreement with a previous report using lung fibroblast CMs to activate HMGB1 production in lung cancer cells [28]. From this study, the small amount of actively released HMGB1 detected under control conditions (with no diffusible factors from fibroblasts) was ascribed to necrotic cell death *in vitro*.

It is well known that monocytes and macrophages can actively secrete HMGB1 in response to various stress stimuli [27]. Although some cancer cells have the ability to secrete HMGB1 into the culture media, these are limited and include colon cancer and malignant mesothelioma [24, 25, 38]. Alternatively, HMGB1 release can be induced by hypoxia [39] or inflammatory cytokines [40]. In breast cancer tissues investigated by immunohistochemistry, it is possible that phosphorylated HMGB1 may reside in the cytoplasm which corresponds to our observations of increased cytoplasmic HMGB1 in breast cancer tissues (data not shown).

HMGB1 is overexpressed in many types of cancer [41–45] including breast cancer [46–48]. In addition to this intrinsic expression, breast cancer cells can be stimulated by factors released from activated fibroblasts to increase their expression of HMGB1. When these cancer cells die after cytotoxic treatment, extracellular HMGB1 is detected in proportion to the levels of intracellular HMGB1. In the present study, MDA-MB-231 breast cancer cells were induced by BCF-CMs (or by Dox) to express high levels of HMGB1.When cell death was induced, HMGB1 could be subsequently released. It can be hypothesized that HMGB1 could then act upon surrounding cancer cells to induce a degree of resistance to chemotherapeutic agents. In support of our findings, extracellular HMGB1 has been shown by Luo et al. to stimulate the regrowth and metastasis of cancer cells that survived prior chemotherapy [29].

Using ‘dead cancer-CM’, our results indicate that HMGB1 may be one component released by dying cells that can influence the response of other cancer cells to Dox. However, the viability of breast cancer cells could not be completely rescued by an HMGB1 neutralizing antibody, implying that other substances released from dead cancer cells may be responsible for Dox resistance. Extracellular HMGB1 binds to the ‘receptor for advanced glycation end products’ (RAGE), in cancers but not normal tissues [49]. However, HMGB1 can promote 3T3 fibroblast wound healing by inducing cell proliferation and migration, and this effect occurs through the activation of the RAGE/MEK/ERK pathway [50]. HMGB1 caused concentration and time-dependent increases of IL-6 production via RAGE, c-Src, Akt, p65, and NF-κB signaling pathways [51]. Although the pathways activated by extracellular HMGB1 in breast cancer cells have not been identified, they may lead to the induction of cancer progression and drug resistance. The ability of released HMGB1 to trigger drug resistance in cancer cells is reportedly due to autophagy [52, 53]. In addition, autophagy can also play a role in anthracycline resistance in triple-negative breast cancer (TNBC) [54]. This is supported by our findings that HMGB1 was linked to autophagy and Dox resistance in TNBC MDA-MB-231 cells.

The data reported here indicate the potential of extracellular HMGB1 released from breast cancer cells to exert a paracrine effect on surviving cancer cells enabling them to resist Dox therapy. An anti-HMGB1 antibody or specific inhibitor (i.e. glycyrrhizin) [55] or targeting its proposed receptors (RAGE and the toll-like receptor 4, TLR4) on cancer cells may prevent or inhibit the development of drug resistance. In contrast, there is the evidence that chemotherapeutic drug-induced HMGB1 can mediate the activation of innate immunity and tumor clearance [56]. Thus caution must be exercised, given the potential positive and negative aspects of HMGB1 expression at different phases of tumor development and during treatment.

## Conclusions

**Figure 7:**
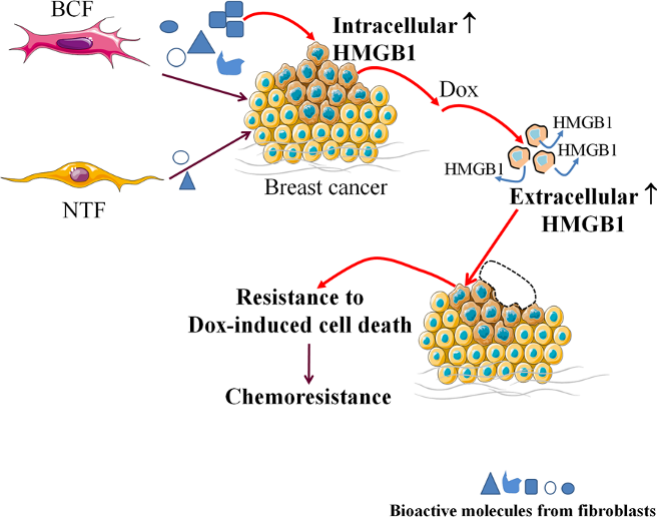
**Schematic diagram illustrating the potential of secreted substances from breast cancer-associated fibroblasts (BCFs) to induce expression of intracellular HMGB1 in breast cancer cells.** After exposure to a chemotherapeutic agent, in this case doxorubicin, (Dox), this increased intracellular HMGB1 can be released and may function in a paracrine manner to induce acquired chemoresistance of the nearby surviving cancer cells.

The findings reported here highlight the potential of cancer-stromal fibroblast interactions to drive chemoresistance in breast cancer in part as a result of fibroblast-induced HMGB1 production and release into the tumor microenvironment with paracrine effects on neighboring cancer cells (Figure 7). To support this hypothesis, circulating HMGB1 levels could be tested as a predictor of responses to neo-adjuvant chemotherapy in breast cancer patients [57]. High levels of serum HMGB1 in patients have been correlated with drug resistance whereas low HMGB1 indicated sensitivity. Targeting cancer-associated fibroblasts which have more genetic stability than cancer cells is an alternative therapeutic approach [30] to interrupt the cycle of fibroblast-induced HMGB1 in mediating acquired chemoresistance.

## Abbreviations

ASMA: Alpha smooth muscle actin
HMGB1: High mobility group box 1
CK: Cytokeratin
CM: Conditioned-medium
Dox: Doxorubicin
BCFs: Breast cancer-associated fibroblasts
NTFs: Non-tumor-associated fibroblasts
RAGE: Receptor for advanced glycation end products.
